# “It seems like they are guessing”: a qualitative study of the prescribing process from the perspective of individuals diagnosed with personality disorder

**DOI:** 10.3389/fpsyt.2026.1850502

**Published:** 2026-08-20

**Authors:** Flavio Di Leone, Jesper Fransson, Malin Sundquist, Jennifer Strand, Sophie I. Liljedahl, Peter Sand

**Affiliations:** 1 Unit for Personality Disorders, Region Västra Götaland, Department of Psychiatry for Affective Disorders, Sahlgrenska University Hospital, Gothenburg, Sweden; 2 Section of Psychiatry and Neurochemistry, Institute of Neuroscience and Physiology, Sahlgrenska Academy, University of Gothenburg, Gothenburg, Sweden; 3 Department of Psychotic Disorders, Sahlgrenska University Hospital, Region Västra Götaland, Gothenburg, Sweden; 4 Department of Psychology, University of Gothenburg, Gothenburg, Sweden; 5 National Specialized Medical Care Unit for Severe Self-Harm Behaviours, Department of Psychiatry for Affective Disorders, Sahlgrenska University Hospital, Gothenburg, Sweden

**Keywords:** personality disorder, pharmachological treatment, prescribing, qualitative study, thematic analysis

## Abstract

**Introduction:**

Psychiatric medications are frequently prescribed for adults with personality disorders (PD), despite clinical guidelines emphasizing psychotherapy as first-line treatment and limited evidence regarding patients' experiences of pharmacological care. This study explored how adults with PD experience the prescription and follow-up of psychiatric medications in outpatient psychiatric services.

**Methods:**

Ten adults with a registered PD diagnosis and experience of prescribed psychiatric medication were purposively recruited from outpatient psychiatric clinics in a metropolitan area in Sweden. Semi-structured interviews explored participants' experiences of medication use, prescribing processes, shared decision-making, and follow-up. Data were analyzed using inductive thematic analysis.

**Results:**

Two overarching themes were identified. Belief in the Perfect Match—and What Happens When It Fails described participants' hopes that medication would reduce emotional instability while raising concerns about losing aspects of their identity or authenticity. Bearing the Whole Burden reflected experiences of prescribing as arbitrary or procedural, limited involvement in treatment decisions, inconsistent follow-up, and a tendency to internalize responsibility when medications did not produce the expected benefits. Participants often perceived medication as a temporary substitute for more comprehensive care rather than as a meaningful therapeutic intervention.

**Discussion:**

Experiences of pharmacological treatment for PD extended beyond symptom management to encompass issues of identity, responsibility, and trust in healthcare. These findings underscore the need for transparent treatment rationales, shared decision-making, and structured follow-up, alongside integration of pharmacological treatment within broader psychosocial interventions. Such an approach may strengthen therapeutic relationships, reduce self-blame, and improve treatment engagement for individuals with PD.

## Introduction

Pharmacological treatment for individuals diagnosed with Personality Disorder (PD) remains a contested and complex domain within psychiatric practice.

Clinical guidelines generally discourage the use of psychiatric medications for the core symptoms of PD, while simultaneously endorsing their use in managing acute distress, as adjunctive treatment, or in the presence of co-occurring psychiatric conditions (1). Although formally sound, these recommendations offer limited practical support for clinicians working in a highly complex clinical reality, where individuals present with severe distress, acute crises, and multiple co-occurring conditions (2). Clinicians describe feeling exposed and insufficiently equipped to address service users’ suffering, resulting in a situation where medication, instead of functioning as a straightforward therapeutic intervention, becomes a source of uncertainty and strain within the treatment process (3).

As a result, pharmacological treatment for individuals diagnosed with PD remains extensive, often characterized by polypharmacy and off-label prescribing (4). In Sweden, only approximately 10% of individuals with personality disorder remain medication-free one year after diagnosis, while one-third are prescribed three or more psychotropic medications concurrently (5). Antidepressants are the most commonly prescribed class, followed by anxiolytics and antipsychotics (6).

Ambivalence and skepticism permeate also how service users experience the process of being prescribed psychiatric medication. While some individuals view medications as a potential source of relief or of emotional stability, others associate them with concerns about adverse effects, long-term use, and a lack of clear therapeutic rationale or personal relevance (7, 8). This skepticism is frequently informed by service users’ own or reported experiences of minimal or negligible meaningful improvement (9). These accounts are consistent with meta-analytic and systematic review findings indicating uncertain efficacy and limited generalizability of pharmacological interventions for both the core symptoms of PD and other co-occurring psychiatric conditions (10, 11). Another commonly reported experience among service users is the perception that medication prescribing contributes to a broader medicalization of their difficulties, functioning as a substitute for interventions they consider more valuable, particularly psychotherapy (12). In this context, service users often perceive the prescription of medications as a way of “buying time,” while prescribers tend to interpret service users´ expectations for relief as a desire for a quick fix (13).

A key issue in the prescribing process for service users diagnosed with PD is the growing evidence that treatment decisions are influenced less by clinical symptom profiles and more by relational and interpersonal dynamics (14). Interpersonal difficulties may alter and complicate how needs are expressed, how information is interpreted, and how shared goals are realized (15). In attempting to navigate interpersonal challenges, clinicians may find themselves using medication in ways that extend beyond its medical purpose, allowing it to function as a tool for alliance building (16). Pressures within the clinical encounter, including strong expectations from service users and concerns about provoking anger or escalating distress on the part of the provider, further influence clinicians’ decisions regarding medication (17). Consequently, challenging interpersonal dynamics may lead to a form of negotiation around pharmacological treatment, which can reinforce service users’ perceptions that behaviors and personal characteristics such as being regarded as difficult, emotionally intense, or resistant may influence the type of treatment they are offered (18).

Taken together, these findings highlight a problematic process unfolding in the context of limited trust and reduced interactive capacity among both clinicians and service users, which may undermine the therapeutic relationship and compromise shared decision-making, ultimately affecting the quality of care for both clinicians and service users. By centering service users’ perspectives, this study seeks to clarify how prescribing is experienced by those most directly affected.

## Materials and methods

This qualitative study aimed to explore how individuals diagnosed with PD experience the prescribing of psychotropic medication. The study was conducted and reported in accordance with the Consolidated Criteria for Reporting Qualitative Research (COREQ), and a completed COREQ checklist is provided as Supplementary Material.

The study was guided by the following research questions:

1. What expectations do individuals diagnosed with PD have regarding pharmacological treatment?
2. To what extent do individuals diagnosed with PD feel involved in decisions to initiate pharmacological treatment?
3. How do individuals diagnosed with PD describe their experiences of transparency throughout the initiation and follow-up of pharmacological treatment?

### Participants and recruitment

Ten adults diagnosed with PD who had experience with prescribed psychiatric medications were recruited from two of the four outpatient psychiatric clinics at a large urban university hospital in Sweden. At the time of the study, each clinic operated as a parallel general psychiatry unit with an integrated personality disorder team holding the same clinical mandate. Participants were drawn from two of these units, ensuring access to service users receiving comparable forms of assessment, treatment, and follow-up.

Inclusion criteria required participants to be aged 18 or older, have a registered diagnosis of PD, and have either previously used or currently be using prescribed psychiatric medication. PD diagnoses were extracted from each participant’s most recent clinician´s entry record. Diagnoses had been established as part of routine specialist clinical assessment and were based on a comprehensive evaluation conducted by at least a psychologist and a psychiatrist. No additional structured diagnostic assessment was conducted for the purposes of this study. At the time of recruitment, diagnoses were recorded according to the ICD-10 classification.

Exclusion criteria included acute psychiatric instability or cognitive impairment that could interfere with informed consent or participation.

Sampling was conducted using an interval-based selection procedure. The sampling frame consisted of 653 individuals, identified through the electronic health records of the outpatient clinics, who met the eligibility criteria between September 2022 and May 2024. Based on a target of inviting approximately 15 eligible patients, the first participant was chosen at random using a random number generator, after which every ninth patient was approached for participation. To facilitate recruitment, the final four participants were recruited consecutively at one of the participating outpatient clinics, where a psychologist invited eligible patients to participate during scheduled follow-up appointments. Following ongoing discussions between the researchers conducting the interviews (JP, JS and MS) throughout data collection and preliminary analysis, recruitment was concluded when the research team judged that the interviews provided sufficient information to adequately address the study aim, in accordance with the principles of information power (19). Those who accepted were then contacted by a researcher (PS) and invited to share their perspectives in an interview regarding their experiences of receiving and following up on prescribed medication as part of their treatment.

Participants received a written information sheet describing the study’s purpose, ethical approval, and procedures, along with a consent form. The informed consent process explicitly emphasized that participation was entirely voluntary, independent of ongoing clinical services, and would have no impact on current or future care. The consent form also clarified that some interview questions could involve recalling and discussing potentially distressing experiences. Participants were informed of their right to decline to answer specific questions and to withdraw from the study at any time without providing a reason and without any consequences for their treatment or relationship with clinical services.

After providing written consent, participants were contacted by the interviewer to arrange the interview, which was conducted at their respective outpatient clinics.

In total, 14 patients were invited, of whom 10 agreed to participate and 4 declined.

### Demographic and clinical characteristics

The demographic and clinical characteristics of the study sample are presented in **Table 1**. The study included 10 participants (8 women and 2 men), ranging in age from 23 to 43 years (M = 32.4 years). All participants had a formal diagnosis of a PD, most commonly Emotionally Unstable Personality Disorder (EUPD). Several participants also had co-occurring psychiatric diagnoses, including Attention-Deficit/Hyperactivity Disorder (ADHD), Cyclothymia, Dysthymia, Generalized Anxiety Disorder (GAD), and Obsessive-Compulsive Personality Disorder (OCPD).

**Table 1:** Clinical and demographic characteristics of the sample.

ID	Sex	Age	Diagnoses	Currently using medication	Years since first prescription
*01*	*F*	*37*	*(EUPD)*	*Yes*	*13*
*02*	*F*	*29*	*(OCPD)*	*Yes*	*5*
*03*	*F*	*29*	*EUPD, ADHD*	*No*	*Unknown*
*04*	*F*	*26*	*EUPD, ADHD*	*Yes*	*14*
*05*	*F*	*42*	*EUPD, Cyclothymia*	*Yes*	*13*
*06*	*F*	*32*	*AUPD, ADD*	*Yes*	*6*
*07*	*F*	*32*	*EUPD*	*Yes*	*12*
*08*	*F*	*23*	*EUPD, Dysthymia*	*Yes*	*2*
*09*	*M*	*43*	*DPD, GAD*	*Yes*	*21*
*10*	*M*	*31*	*(NPD), ADHD*	*Yes*	*8*

APS, Avoidant Personality Disorder; GAD, Generalized Anxiety Disorder; ADHD, Attention-Deficit/Hyperactivity Disorder; ADD, Attention Deficit Disorder; EUPD, Emotionally Unstable Personality Disorder; NPD, Narcissistic Personality Disorder. OCPD, Obsessiv-compulsive personality disorder; DPD, Dependant PD.

At the time of the interview, 9 of the 10 participants had current prescriptions for psychiatric medication. The length of time since first being prescribed medication varied substantially, ranging from 2 to 21 years (Median = 12 years), reflecting both early and long-standing experiences of pharmacological treatment within the group. .

### Data collection

Data were collected through semi-structured, in-depth interviews conducted face-to-face by two clinicians (JF, MS), who were residents in psychiatry at the time of data collection and were supervised in qualitative interviewing. Interviews lasted approximately 45 to 90 minutes and were audio-recorded and transcribed verbatim. Transcripts were de-identified, and data were stored securely to ensure confidentiality.

The interview guide was developed by two of the authors (PS and FDL; **Supplementary Appendix 1**). Informed by the reviewed literature and their clinical experience, the interviews explored participants’:

- expectations prior to medication prescription
- information received about expected efficacy, side effects and follow-up plan
- involvement in treatment decision-making
- overall attitudes toward medication

### Data analysis

Data were analyzed using inductive thematic analysis, following the six-step process outlined by Braun and Clarke (20). Given the concrete nature of the content and the straightforwardness of participants’ responses, an essentialist, experience-focused approach was applied in both coding and theme development (21).

NVivo software (version 14) was used to facilitate data management and coding. The initial coding process was performed independently by two researchers (FDL, JS). After completing their initial coding of three interviews separately, they met to compare, refine, and reconcile their interpretations. FDL then continued the coding of the remaining transcripts, grouping codes and developing an initial structural map to represent relationships among them. The analysis generated 194 meaning units, which were subsequently condensed into 42 distinct codes. Theme development was carried out by all authors (FDL, JS, SIL, and PS) in three collaborative meetings. After each meeting, further abstraction was undertaken, and codes were reorganized across subthemes to improve conceptual clarity and coherence.

Throughout the analytic process, themes were repeatedly checked against the raw data to ensure fidelity. The team also engaged in ongoing reflexive dialogue regarding how their respective professional backgrounds—psychiatric, psychological, and academic—shaped their interpretations and the evolving thematic framework.

### Reflexivity and positionality

The research team represented a range of professional and theoretical backgrounds, which enriched the analytic process and interpretation of findings. One author (FDL) is a practicing psychiatrist with clinical experience in diagnosing and treating PD and prescribing psychiatric medications. The other researchers (PS, JS, and SIL) are psychologists, academic and clinical researchers with complementary clinical backgrounds and theoretical orientations.

All interviewers were clinicians who had worked across a variety of psychiatric settings involving pharmacological interventions. None worked at the participants’ respective outpatient services at the time of data collection. This ensured both the clinical competence needed to navigate the topics with confidence, while also avoiding any direct or indirect therapeutic relationship that might have influenced participants’ responses. The interview transcripts showed no evidence of a medicalized or hierarchical dialogue; participants appeared comfortable and forthcoming, expressing their views on medication and treatment openly and in their own terms.

Because the researcher responsible for recruitment (PS) held a managerial position at one of the outpatient clinics, the initial recruitment process led to an unintended overrepresentation from that unit. Six of the ten participants were drawn from the clinic where PS worked, highlighting a potential imbalance and the practical difficulties of recruiting in a context where the researcher is part of the hierarchy. To address this, additional service users from other outpatient units were invited, which broadened the recruitment base and reduced the risk that participation was influenced by PS’s managerial role rather than genuine willingness to take part.

### Ethical considerations

The study was approved by the Swedish Ethical Review Authority (Dnr 2020-07105).

Participants received written and oral information about the study and provided written informed consent.

Given that participants were recruited within psychiatric services and given the documented experiences of people with PD finding healthcare to misunderstand and invalidate them, particular attention was paid to potential vulnerabilities and power asymmetries (22). To reduce implicit pressure to participate, it was emphasized that the research was independent of clinical decision-making and that interviewers were not involved in participants’ treatment.

Interviews addressed experiences of psychiatric medication that could evoke distress. Participants were reminded that they could pause or terminate the interview at any time, and time was allocated for debriefing when needed.

Finally, issues of interpretive authority were considered. Reflexive discussions were therefore integrated throughout the analytic process to critically examine preconceptions and to avoid re-pathologizing or dismissing participants’ perspectives. Ethical considerations were thus approached as an ongoing, reflexive process rather than solely a procedural requirement.

## Results

The analysis generated two overarching themes and six subthemes that together illustrate how participants experienced the prescription of psychiatric medication (**Table 2**).

**Table 2:** Themes and subthemes derived from interviews with participants diagnosed with personality disorders on their experiences of the prescribing process.

Theme	Subtheme	Description
1. Belief in the Perfect Match – and What Happens When It Fails	1a. Anticipation of Relief and Balance	Hope that medication would restore emotional stability, reduce reactivity, and help participants feel like themselves again.
	1b. Disillusionment When Expectations Fall Short	Frustration, uncertainty, and doubt when medication effects did not meet expectations.
	1c. Shouldering the Blame	Internalizing responsibility for lack of improvement, often reinforced by clinical interactions.
2. Bearing the Whole Burden	2a. When Treatment Feels Arbitrary	Prescribing perceived as random or non-individualized, undermining trust.
	2b. Feeling Tokenized in Decisions	Participation in decision-making experienced as superficial or disregarded.
	2c. Left to Bear Responsibility Alone	Participants felt responsible for managing treatment, monitoring effects, and advocating for care.

### Theme 1. Belief in the perfect match – and what happens when it fails

This theme reflects participants’ belief in finding the “perfect” medication and how this belief shaped their expectations, emotional responses, and sense of responsibility within the prescribing process. When medication felt attuned to their inner experience, it brought balance, stability, and a sense of returning to themselves. This alignment, however, was fragile. For some, benefits were inconsistent or diminished over time, leading to frustration, doubt, and emotional exhaustion. When improvement failed to materialize, disappointment often turned inward; participants questioned their own adequacy and felt personally responsible for the lack of progress.

#### Subtheme 1a. Anticipation of relief and balance

This subtheme captures how participants’ medication-related reasoning was primarily driven by an anticipation of relief rather than explicit expectations of outcomes. While anticipation and expectation are closely related, participants described anticipation of relief as the motivating force behind their willingness to try medications, reflecting the underlying “why” of their medication inquiries. This anticipation was often accompanied by considerations of balance, weighing hoped-for relief against potential costs or consequences.

Participants described a strong belief in the “right” medication, which could restore a sense of emotional stability and personal coherence after long periods of internal chaos. As one participant explained, medication promised a form of help that felt both accessible and achievable:

When the treatment actually works, it feels really good. It feels like you’re actually doing something and making progress. And it’s quite simple too, just taking the medication, compared with having to do tasks or other things you would otherwise need to manage (Participant 2).

For medications to provide meaningful relief, participants thought they must offer both a reduction in emotional reactivity and a restoration of emotional availability. For some, this meant regaining a sense of control over heightened sensitivity and intense emotional responses, allowing them to engage with their feelings without “letting it run off the rails completely” (Participant 5).

As one participant described it:

*I think it affects me, even though I don’t know exactly how. For example, when I watch a scary movie, it doesn’t feel as frightening anymore, and I’m not as afraid of the dark. I don’t know if it’s really because of the medication or if I’m just imagining it, but I still believe it makes a difference. I experience things differently now. At the same time, it’s part of my difficulties, that I’m so sensitive, and that sometimes it becomes a problem for me (Participant 8).*

For others, the medications allowed previously muted emotions to resurface, even if their overall emotional reactivity remained partly unresolved:

“*It was the first time I could get in touch with certain feelings again, and I realised: my god, I haven’t laughed in I don’t know how long. I’ve been on the medication for maybe three years, and it was an enormous difference. I had been very emotionally shut off. It saved me in some situations, but in many others it wasn’t helpful at all.*” *(Participant 10).*

Medication also reshaped emotional attunement in quieter, everyday ways. Participant 1 explained that they had become comfortable being alone and even appreciated becoming “a pretty boring person and actually liking it”:

“*I used to struggle a lot with being alone and constantly needed people around me. Now, I enjoy my own company. It feels like a change in my personality, but in a positive way.*”

This newfound equilibrium often extended to social belonging:

“*I often used to feel like I was in the way, that people found me bothersome … But since I started taking [medication], I feel that much less. I feel more like I have a right to be here. When I swim, I used to think I was doing it wrong, in the wrong lane or swimming the wrong way. Now it feels more like, if I end up in the wrong place, it doesn’t matter—I just move … It’s hard to explain, but it’s largely about not feeling like a burden to the world anymore*” *(Participant 1).*

#### Subtheme 1b. Disillusionment when medications fall short

This subtheme centers on participants’ experience of relief that failed to match what they had hoped for. Although symptoms were sometimes reduced, the relief was described as coming at a personal cost, a trade-off that could appear to serve others more than the patient.

Even in improvement, there was a sense of negotiation between who they were, who they became on medication, and who they hoped to be. Across accounts, anticipation of relief was intertwined with vulnerability.

“*It may sound tragic, but when I take the medication, it feels like I can stand myself. I don’t do or say things I later regret. At the same time, it feels strange. I remember the nurse said that sometimes you need to get used to the person you become when taking this type of medication. That’s true – I’ve felt a bit like I’ve lost part of my personality. But overall, I still think the medication helps*” *(Participant 3).*

Others reported modest improvements offset by side effects, leading to an overall sense of stagnation:

“Maybe I’ve been in a better mood and more social, but with the side effects it becomes plus minus zero” (Participant 2).

Ultimately, the changes were experienced less as an internal shift and more as something that made them easier to relate to those around them:

“I think I became easier for people around me to handle … the feelings are still there, but their expression is very minimal” (Participant 9).

The turning point for many was when initial improvements faded, fostering a growing sense of disillusionment and even mistrust. One participant recalled that

“the effect was strongest in the beginning, then it flattened out” (Participant 1),

while another described how a promising start quickly stalled despite dose increases:

“It felt better at first … but then it stopped working. Even when they raised the dose, nothing happened” (Participant 5).

Another described repeatedly trying to evaluate whether the medication was doing anything at all:

“The more I think about it, the more I wonder: has this actually helped, or not?” (Participant 8).

Holding onto the belief that medications could “fix” what felt fundamentally wrong inside, many embarked on a search for the perfect match that in the end deepened into hopelessness:

“I had very high expectations … that you take pills and get saved…. I’ve tested maybe twenty different medications … If one didn’t help, they added another” (Participant 4).

This sometimes left participants feeling pressured toward stronger medications without understanding why:

“It feels a bit scary, thinking I need the strongest medications, even though I don’t know why I should feel that way” (Participant 10).

Ultimately, the anticipation itself transformed into a question of permanence:

“What if they work—does that mean I have to take them for the rest of my life?” (Participant 10).

#### Subtheme 1c. Shouldering the Blame

For many participants, disappointment with medication gave rise to a pervasive sense of personal responsibility for its perceived failure. The lack of noticeable progress felt quickly reverted as accusation, internalized and attributed to the patients personally:

“I feel it simply doesn’t help, and when someone says it should, I think there’s something wrong with me for it not working. Sometimes I wonder if I’m just imagining that it doesn’t work” (Participant 1).

Even when hope persisted for finding the “perfect” medication, discouraging and dismissive communication from clinicians framed the patient as the problem rather than recognizing the limitations of the treatment:

“*‘This isn’t working either; she’s probably an impossible case.’ That’s the kind of thing I’ve heard—that they’ve tried everything possible. At the same time, there are still medications left to try, but it still feels like: ‘No, this doesn’t work either. What are we going to do now?’ That’s roughly how the follow-ups have felt. I don’t remember much else of what they said*” *(Participant 4).*

Since many reported concurrent trials with medications, each consultation carried the tension of prior disappointments, misaligning the communication:

“When it hasn’t worked so well, I’ve talked to my doctor about it. Sometimes there’s a certain frustration … And when I’ve had my own thoughts and reflections, it hasn’t really felt like they’ve taken them on board” (Participant 1).

Even routine adherence carried undertones of blame, and the ritual of taking medication became both necessary and shame-laden:

“I barely took any vitamins before, so it feels a bit strange now … At the same time, I feel almost like a [recreational] drug user … but I know I might actually need them, unfortunately” (Participant 10).

The limited acknowledgment of participants’ experiences and concerns left them with a persistent sense that treatment failures were their own responsibility, as one reflected:

“It has made me feel all the time that it’s my fault that I can’t taper off the medication” (Participant 9).

When neither symptoms nor clinical conversations offered a sense of direction, the entire cycle of choosing, taking, and evaluating medication began to feel devoid of meaning. As one participant put it,

“Right now it feels like the medication is just there, without any real purpose. I still feel unwell … and I honestly have no idea anymore why I’m taking it” (Participant 9).

### Theme 2. Bearing the whole burden

The second theme reflects the external journey of navigating the prescribing process. Participants described the system as a largely superficial or procedural process, in which decisions, follow-up, and understanding often felt opaque or delegated to them without sufficient support. Rather than experiencing collaboration or guidance, they were left to manage the practical and emotional weight of treatment largely on their own, carrying responsibility for outcomes without the corresponding authority or insight. This theme highlights how the structure and dynamics of care can leave patients feeling exposed, unsupported, and burdened by the very system intended to help them.

#### Subtheme 2a. Treatment feels arbitrary

Participants frequently described the prescribing process as inconsistent and circumstantial. No participant reported experiencing clarity in the prescribing process, and accounts varied considerably between participants, within the experiences of the same participant, and even regarding the same medications. Participants lamented the lack of a more considered and personalized approach, highlighting the importance of

“…a more in-depth conversation with the doctor about the medication and why they think it would suit me” (Participant 2).

Decisions about medication and follow-up were therefore perceived as being driven more by the prescribers’ impressions, preferences, and working environment.

“It’s so easy to just prescribe medication, and I think many clinicians do exactly that. Like, ‘I like this drug, let’s give it to everyone.’ That’s pretty much how it feels with what I’m on now, even though a lot of people seem to be on the same thing. And sure, if it works, great. But still…” (Participant 1).

The repetitive trial and error that shaped the prescribing process also reinforced the sense of randomness in the experience of care:

“Since I’ve tried so many different types of medication, sometimes it feels like they’re just guessing” (Participant 3).

But the perception of arbitrariness transpires even when participants felt genuinely heard:

“A lot depends on the doctor and how they ‘sell’ the medication [laughs]. If you trust the doctor, feel they really listen and understand how I feel, and they say: ‘I think this might help you, not just because it’s my favorite medication—try this,’ then it works better” (Participant 10).

For other participants, prescribing felt dictated by circumstances far from their actual needs. Medication became a “last resort,” something clinicians turned to when other options seemed burdensome, unavailable, or were prematurely dismissed, which only reinforced the sense of arbitrariness and incongruity in the experience of care. Some described clinicians as rushing toward medication in an unclear and unsettling way:

“It felt like they wanted to start with medication first and then investigate further to see what other type of treatment might suit me” (Participant 4).

Some noted the emphasis on pharmacological treatment as overshadowing other forms of care: “I’ve felt that there’s often an excessive focus on medication, which means other types of treatment end up receiving very little attention” (Participant 7). Consequently, medications were often experienced as a placeholder rather than a meaningful or intentional intervention:

“I contacted care and said I needed conversation, that I needed to talk. But it was really hard to get someone to talk to me. And then it became more like: ‘Here, take medication and see if it works.’ As if I was supposed to have something while waiting for other care. It feels a bit like the medication is just something you get while you wait” (Participant 2).

Arbitrariness cultivated a quiet sense of inevitability, as medication came to symbolize both personal and systemic failure: “During most of the first year, I chose not to take medication. I preferred that they do more in other ways. But when I felt so unwell that I was hospitalized, I started taking medication because it felt like I had no other option” (Participant 9).

#### Subtheme 2b. Decision-making is tokenized

Most participants reported some involvement in decision-making, but the process often left them with lingering confusion and doubt, giving the impression that their participation was largely nominal or superficial.

“I’ve taken many medications over the years, and I agreed to all of them. But maybe it’s more that I’ve believed this is just how it’s supposed to be, how treatment works. For that reason, I’ve felt that it was okay, felt right, and felt involved in the process. I’m not really sure.” (Participant 5)

Several described that they felt involved in the decision making just to see their concerns dismissed.

“Sometimes you can even notice it in their tone, a little sigh: ‘Yes, but we’ll do it this way anyway’” (Participant 3).

Many felt this happened because their individuality was overlooked in favor of diagnostic stereotypes. When participants were primarily addressed as a diagnosis rather than as individuals, their concerns were more readily dismissed in the prescribing process, contributing to a sense of having to carry the consequences of treatment decisions largely on their own:

“I’ve gotten quite angry at the doctor many times, and several times I’ve been told: ‘This is very typical for your diagnosis, that you get angry [when you disagree].’ It has felt like my feelings have been brushed aside.” (Participant 2)

This uncertainty, amplified by a fragile sense of identity and emotional stability, shapes the therapeutic relationship as insecure and threatening:

“I wish there was some way to confirm what’s happening—whether it’s me getting swept up in my emotions or if something actually went wrong in how I was treated. I just can’t tell the difference, and that uncertainty is exhausting.” (Participant 10).

At times, the sense of tokenistic involvement extended to the tension between being treated as an adult and yet being given overwhelming or inappropriate detail:

“I would probably say I was a little too involved. I was told far too much about myself [at Child psychiatric services]—things that really should have gone to my mother. I should have been part of the decisions, but I didn’t need to hear all the details about myself that then stuck in my head” (Participant 9).

In the end, tokenization fails and the need for a more clear and reciprocal communication about medication burst out: “*I need a proper review of my medications, where I can discuss ideas without it being immediately decided for me*”, lamented a participant, which later wishes expressed the wish

“to be seen as a whole person, not just a collection of diagnoses” (Participant 5).

#### Subtheme 2c. Bearing responsibility alone

Across accounts, participants described a persistent sense of carrying the practical, emotional and evaluative burden of their own treatment.

Early promises about short-term medication use often contrasted sharply with reality, leaving some with a retroactive sense of having been misled and a present-day responsibility for which they felt unprepared. As expressed by one participant:

“When I was 13 they told me I would be on this medication for at most a year. Now I’m 26. I’m still quite angry that I didn’t push to start tapering back then” (Participant 9).

This burden of responsibility for taking, managing and evaluating their own medication was intensified by uncertainty about how much initiative they were expected to take in the context of treatment. Many felt responsible for suggesting treatments or arriving with fully formed preferences, despite lacking the expertise or energy to do so.

“I’m unsure how much I’m supposed to push for them to find something better … I want to look things up myself, but you can only manage that sometimes as a patient. The difficult part is that I have to know what’s wrong, what I need, and then explain it to the doctor so I get the right help. It’s very hard to formulate all that myself” (Participant 2).

Others echoed the same dynamic in simpler terms: “Sometimes it feels like it’s up to me to figure out what I want to try, and that’s hard because I don’t actually know anything!” (Participant 8).

Limited contact with clinicians also pushed participants toward external sources of information and validation, sometimes with troubling effects. Descriptions of online medication cultures revealed competitive, identity-driven dynamics that shaped how young people perceived their own suffering.

“People scare each other and it becomes some kind of competition … If you didn’t take the ‘right’ meds, you weren’t really sick … It becomes an effect of not seeing a doctor often enough. Then this comparison behavior develops, especially among us with borderline—as if you have to prove how unwell you are to get the right help” (Participant 1).

The expectation to self-monitor and self-assess further added to the sense of being left alone with responsibility. Participants noted that frequent questions such as “*better or worse?*” created pressure to quantify states that felt subjective and unstable.

“The more you look and measure, the more unsure you get. It can even become a bit dangerous … it creates more fixation and worry” (Participant 10).

While many tried to keep informal track of their symptoms, motivation dropped when their efforts were not acknowledged:

“No one asked to see the first diary I wrote, so I don’t feel motivated to keep another log that might never be used” (Participant 3).

Decision-making demands also strained self-confidence. Some participants explicitly stated that they could not make complex treatment decisions on their own, yet were sometimes met with an overload of options rather than guided support:

“It crushes your confidence … sometimes they understand, but sometimes they mean well and give many alternatives and questions that just stress me” (Participant 10).

Finally, the responsibility extended beyond decisions about medication to the emotional labor of maintaining continuity in care. Difficulty accessing a new clinician could leave patients feeling trapped between frustration and fear of abandonment:

“I wanted to change doctor … it was promised several times, but it never happened. After a while so much time had passed that I didn’t dare switch, afraid of ending up alone … so I still have the same doctor, and despite everything I appreciate it because he has been with me the whole time” (Participant 1).

## Discussion

Being prescribed psychiatric medication emerged in this study as far more than a clinical transaction. Rather, it became a context in which questions of identity, responsibility, and relational positioning collided. Medications carried hopes of emotional stabilization, but when effects were unclear or insufficient, participants often interpreted this as personal failure or dysfunction. Simultaneously, limited transparency, inconsistent follow-up, and unclear rationales for prescribing contributed to feelings of arbitrariness and isolation.

Several participants described internalizing doubt about their own perceptions and feeling subtly blamed when treatment did not produce the expected results. At the same time, they sensed that clinicians sometimes viewed their reactions or uncertainties as personal shortcomings rather than meaningful information. This dual pressure created a heavy emotional load in which participants held themselves responsible for both their symptoms and the success or failure of the medication. In turn, this shaped how they engaged with treatment and how they understood themselves: not simply as patients taking medication, but as people navigating a system where clinical decisions carried implications for identity, credibility, and responsibility. Across the interviews, one pattern was the intense anticipation of relief. Medication was often framed as the hoped-for escape from emotional reactivity and instability, whether directed inward or outward. Participants described waiting for concrete, measurable signs that something real was changing. Without such visible evidence, they struggled to interpret their experiences and often concluded that they themselves were the problem. This was especially pronounced when the imagined effect of medication resembled a fundamental transformation of one’s identity. If that transformation did not occur, some interpreted the outcome not as a limitation of the medication but as confirmation of personal defectiveness, inadequacy, or even impossibility.

Layered onto this was the ongoing struggle with agency. Participants expressed the desire to be responsible for their own improvement while simultaneously needing to trust that the clinician could understand and use their experiential information in a meaningful way. This balancing act was fraught. Too much responsibility felt like being set up to fail; too little felt like losing authorship of one’s own life. The result was an oscillation between self-blame and self-doubt, between reaching for control and relinquishing it in the hope that someone else could interpret their needs more accurately than they could themselves (23, 24).

However, the prescribing process introduced additional complexities beyond the intrapersonal. Many of the difficulties described were embedded in the structural and procedural realities of mental health care. Limited availability of psychosocial treatments, brief consultations, and a tendency to lean on medication as the default response meant that prescriptions were often experienced as a placeholder rather than a thoughtfully integrated intervention. When other forms of support were inaccessible, participants sometimes interpreted the reliance on medication as a sign of therapeutic exhaustion or personal bias. Peer expectations, in the form of online communities, occasionally intensified this dynamic, with others’ experiences acting as a catalyst for trying or abandoning treatments. In this context, prescribing could come to symbolize system constraints rather than shared clinical reasoning, deepening feelings of resignation or helplessness.

These two layers of difficulty – the vulnerabilities tied to identity instability and the process-driven shortcomings of psychiatric services – converged to heighten uncertainty and emotional strain. Yet the findings also show that these difficulties can be mitigated. Participants consistently emphasized that clear explanations, predictable follow-up, recognition of their lived experience, and a stable, collaborative relationship with the prescriber made a decisive difference. When clinicians articulated the purpose of the medication, acknowledged the patient’s subjective expertise, and situated pharmacological treatment within a broader, coherent care plan, patients reported feeling safer, more grounded, and more able to understand and navigate their own reactions.

Taken together, the results highlight that prescribing is not just a technical act but a relational and identity-shaping process. Thoughtful, transparent, and context-sensitive prescribing has the potential to counteract both personal vulnerabilities and systemic limitations, functioning as a therapeutic intervention in its own right (25, 26).

### Clinical implications

These findings point to several practical steps for improving psychiatric prescribing for individuals with identity disturbance, emotional instability, and difficulty navigating care.

First, clinicians may need to provide clearer and more consistent explanations for medication choices. It is well known that even brief, focused rationales can reduce the sense of arbitrariness and help patients avoid interpreting prescriptions as stigmatizing and improve both adherence and response (27). Interestingly, none of the participants spontaneously recalled being informed about off-label prescribing or receiving clear statements regarding guideline recommendations for pharmacological prescription in PD; such knowledge was reported only as second-hand information, based on others’ personal experience or hearsay. While this may partly reflect miscommunication within challenging interpersonal contexts, it also aligns with previous evidence suggesting that prescribers do not consistently rely on clinical guidelines when organizing or deciding on treatment (16). As established in other interventions, communication with individuals with PD is challenging but most effective when it combines trust with factual, stable, and practical information, while also anticipating flexibility in how treatment may unfold (28). In this context, framing emotional stability as a treatment goal—measured in terms of everyday functioning and balance—provides a concrete and understandable basis for prescribing decisions, dialogue about expectations, and setting realistic treatment outcomes. Similarly, addressing personality change as a potential effect or side effect of medication responds directly to a core patient concern, helping to reduce uncertainty and fear. Taken together, these insights offer promising guidance for fostering a more meaningful, transparent, and collaborative dialogue between clinicians and patients regarding medication use.

Second, predictable follow-up structures are essential for patients who struggle with motivation, self-monitoring, or interpersonal stress. Routine check-ins can prevent treatment drift and reduce the sense of being left alone with complex decisions, functioning as both clinical oversight and emotional containment. In this context, individual personality traits, understood as characteristic patterns of emotional and behavioral expression, can guide the organization of follow-up (29). In this study, participants with features of borderline patterns appeared to prefer more active, guided follow-up, whereas those with more internalized traits expressed a preference for more automated or structured systems, consistent with findings from studies exploring adherence across personality types (30).

Third, across all participants, there was a clear desire for follow-up that extended beyond simply prescribing medication, emphasizing that prescribing should be embedded within a supportive, continuous care framework. Clinicians should therefore acknowledge that medications also carry symbolic meanings. Ambivalence may reflect less the pharmacological effect and more what the medication represents: illness, loss of control, or the absence of psychosocial support. Attending to these interpretations can prevent understandable hesitation from being misread as noncompliance. Appreciating service users’ subjective realities is essential to counter the persistent assumption that individuals diagnosed with PD are fully in control of their symptoms or inherently “manipulative,” a view that risks reducing clinical complexity to moral judgment rather than professional understanding (31).

These findings underscore the importance of strengthening training in the relational aspects of prescribing (32). Educational interventions have been shown to improve clinicians’ attitudes and practices toward individuals diagnosed with PD; however, to our knowledge, none have specifically addressed psychotropic prescribing or evaluated prescribing practices as an outcome (33). Interestingly, such interventions are typically offered as optional continuing education rather than integrated into undergraduate or residency training. Framing personality disorder as requiring exceptional expertise may inadvertently reinforce stigma and therapeutic avoidance, whereas integrating these competencies into standard psychiatric training emphasizes that relationship-centered prescribing is a fundamental aspect of good psychiatric care (34).

Finally, prescribing should be embedded within broader, multimodal treatment pathways. Regardless of whether psychosocial interventions are readily accessible, medication should not be offered as a stand-alone treatment, as this risks it being experienced as a placeholder rather than a targeted intervention (Confue, Maidment, & Jones, 2025). This approach may reflect a diagnostic perspective that treats different symptoms or conditions in the same individual as separate “co-morbidities” rather than as interconnected expressions of distress (35). Conceptualizing these conditions as influencing one another could support more integrated approaches that consider personality and symptomatology together (36). Embedding prescribing within psychotherapy, collaborative care, or stepped-care models can help align treatment decisions more closely with patient needs and lived experiences (37).

### Limitations and future directions

Several limitations should be considered when interpreting these findings. First, the study included a small sample of 10 participants from a single outpatient setting without information about demographic characteristics outside of age and sex, which may limit the transferability of results to other clinical contexts and to comparison samples of people receiving treatment outside of this setting. Second, most participants had a diagnosis of emotionally unstable personality disorder (EUPD), which means that the experiences reported may primarily reflect the perspectives of this subgroup and may not fully capture the diversity of experiences among patients with other personality disorder diagnoses. Third, participants were self-selected, introducing the potential for selection bias, as those with particularly strong opinions or experiences regarding medication may have been more likely to participate. Finally, the cross-sectional design captures experiences at a single point in time, which may not reflect how perceptions of treatment and prescribing processes evolve over longer periods.

Future research should extend the limits of this work by including larger and more diagnostically diverse samples and by adopting longitudinal designs to capture changes in experiences and outcomes over time. Qualitative studies conducted across primary care, secondary care, and inpatient settings would help clarify how contextual factors shape the experience and practice of prescribing. Further investigation is also needed into how the ICD-11 classification of PD influences how patients’ needs are expressed and responded to, particularly when stratifying by severity and trait domains (38). In addition, future research should shift attention from *what* is prescribed to *how* prescribing unfolds in clinical encounters, with greater emphasis on relational and communicative processes. Empirical studies are warranted to evaluate prescribing models informed by pharmacopsychological and relational perspectives, including their effects on patient and clinician satisfaction as well as prescribing patterns.

## Data Availability

The raw data supporting the conclusions of this article will be made available by the authors, without undue reservation.
