# Supplementary material for: “It seems like they are guessing”: a qualitative study of the prescribing process from the perspective of individuals diagnosed with personality disorder
Source: Front Psychiatry. 2026 Aug 20;17:1850502. doi: 10.3389/fpsyt.2026.1850502 (PMC13539884; doi:10.3389/fpsyt.2026.1850502)
Supplement: Supplementary file 1 [file DataSheet1.pdf]

COREQ (Consolidated Criteria for Reporting Qualitative Research) Checklist

Paper:

"It seems like they are guessing": A Qualitative Study of the Prescribing Process from the Perspective of Individuals Diagnosed with Personality Disorder

Topic	Item No.	Guide Questions/Description	Reported on
**Domain 1: Research team and reflexivity**			
Personal characteristics			
Interviewer/facilitator	1	Which author/s conducted the interview or focus group?	Data Collection
Credentials	2	What were the researcher's credentials? E.g. PhD, MD	Title page
Occupation	3	What was their occupation at the time of the study?	Data analysis, Reflexivity and Positionality
Gender	4	Was the researcher male or female?	Not reported
Experience and training	5	What experience or training did the researcher have?	Data analysis, Reflexivity and Positionality
Relationship with participants			
Relationship established	6	Was a relationship established prior to study commencement?	Reflexivity and Positionality
Participant knowledge of interviewer	7	What did participants know about the researcher?	Reflexivity and Positionality
Interviewer characteristics	8	Characteristics reported about interviewer/facilitator?	Reflexivity and Positionality
**Domain 2: Study design**			
Theoretical framework			
Methodological orientation and theory	9	Methodological orientation underpinning the study?	Data Analysis
Participant selection			
Sampling	10	How were participants selected?	Participants and Recruitment
Method of approach	11	How were participants approached?	Participants and Recruitment
Sample size	12	How many participants were in the study?	Demographic and clinical characteristics
Non-participation	13	How many refused or dropped out? Reasons?	Participants and Recruitment
Setting of data collection	14	Where was data collected?	Participants and Recruitment
Presence of non-participants	15	Anyone else present?	No
Description of sample	16	Important characteristics of the sample?	Demographic and clinical characteristics

Data collection			
Interview guide	17	Questions/prompts/guides provided? Pilot tested?	Data Collection, Appendix 1
Repeat interviews	18	Were repeat interviews carried out?	Data Collection
Audio/visual recording	19	Audio or visual recording used?	Data Collection
Field notes	20	Field notes made?	Data Collection
Duration	21	Duration of interviews/focus groups?	Data Collection
Data saturation	22	Was data saturation discussed?	Participants and Recruitment
Transcripts returned	23	Were transcripts returned for comment/correction?	-
**Domain 3: analysis and findings**			
Data analysis			
Number of data coders	24	How many data coders coded the data?	Data Analysis
Description of coding tree	25	Description of coding tree?	Data Analysis
Derivation of themes	26	Themes identified in advance or derived from data?	Data Analysis
Software	27	Software used to manage data?	Data Analysis
Participant checking	28	Participants provided feedback on findings?	No
Reporting			
Quotations presented	29	Participant quotations presented?	Results
Data and findings consistent	30	Consistency between data and findings?	Discussion
Clarity of major themes	31	Major themes clearly presented?	Results
Clarity of minor themes	32	Description of diverse cases/minor themes?	Results

Developed from: Tong A, Sainsbury P, Craig J. Consolidated criteria for reporting qualitative research (COREQ): a 32-item checklist for interviews and focus groups. *International Journal for Quality in Health Care*. 2007. Volume 19, Number 6: pp. 349 – 357
