# Supplementary material for: “It seems like they are guessing”: a qualitative study of the prescribing process from the perspective of individuals diagnosed with personality disorder
Source: Front Psychiatry. 2026 Aug 20;17:1850502. doi: 10.3389/fpsyt.2026.1850502 (PMC13539884; doi:10.3389/fpsyt.2026.1850502)
Supplement: Supplementary file 2 [file SupplementaryFile1.docx]

**Appendix 1 – Interview Guide**

1. What diagnosis have you received?
2. What do you know about medication treatment for this diagnosis?
3. Can you describe your experience of being treated with medication?
4. What do you see as the advantages of medication treatment?
5. What do you see as the disadvantages of medication treatment?
6. What side effects have you experienced during or after medication treatment?
7. Were you offered any other psychological treatments?
8. When you first sought care, did you consider medication treatment as an option?
9. What was your attitude toward medication treatment at that time?
10. Did you and your doctor discuss medication treatment for your diagnosis? What do you remember from that conversation?
11. How were decisions about medication treatment made? Did you feel included in the decision-making process?
12. Which medications were you prescribed, and what expectations did you have?
13. What did follow-up with your doctor look like after the medication was started?
14. Did the medication treatment meet your expectations?
15. What information did you receive about medication effects and side effects?
16. How have the medications affected you personally and/or socially?
17. During follow-up appointments, what kinds of questions did your doctor ask?
18. Did the medication treatment work for you? Did you ever feel the need to adjust the medication on your own? Why?
19. Can you give examples of situations where the treatment helped you or affected you negatively?
20. What do you do if you are not satisfied with the medication treatment? (For example, do you contact your doctor, seek another doctor, go to the emergency department, stop the treatment, or look for information on social media?)
